# The association of anthropometric indices and resistant hypertension among type 2 diabetes: a case study of patients attending Kisii Teaching and Referral Hospital, Kenya

**DOI:** 10.4314/ahs.v23i1.56

**Published:** 2023-03

**Authors:** Mary Mueni Mbithi, Tabitha Wanjau, Gordon Nguka

**Affiliations:** 1 School of Health Sciences, Kisii University, P.O.Box 408-40200, Kisii, Kenya; 2 School of Public Health, Biomedical Sciences and Technology, Masinde Muliro University, Private Bag, Kakamega, Kenya

**Keywords:** Resistant and conventional hypertension, type 2 diabetes

## Abstract

Resistant hypertension has been identified as common among type 2 diabetics. Importantly, diabetes prevalence has been increasing in the recent past indicating a future rise in the frequency of resistant hypertension. This study determined the association of anthropometric indices as determinants of resistant hypertension compared to conventional hypertension among type 2 diabetes patients attending Kisii teaching and referral hospital (KTRH). A descriptive study design was used where 96 adult cases with type 2 diabetes were consecutively selected into the study, of which 48 had conventional hypertension and the other 48 participants had resistant hypertension for the last one year. Statistical analysis involved Pearson's Chi-Square test analysed categorical variables while binary logistic regression and odds ratio was used to determine the relationship and association of the study variables. The study results indicated that there is an association between Body Mass Index (BMI) (P=0.014) and resistant hypertension among type 2 diabetes patients attending KTRH. However, the Waist-Hip ratio (P=0.393) was not associated with resistant hypertension among type 2 diabetes patients attending Kisii Teaching and Referral Hospital. These results expanded the understanding of risk factors of resistant hypertension among type 2 diabetics. Therefore, this study recommends comprehensive health promotion programs to encourage and promote lifestyle modification.

## Introduction

Resistant hypertension is a condition that has gained a lot of attention in recent years. It has been realized as an important and continuously growing subset of the hypertensive population, and data showing the associated risk factors in this group of patients are limited, more so among the diabetes patients^1^.

The anthropometric indices as determinants of resistant hypertension have not been well studied and remain unknown in the Kenyan population. Despite the fact that diabetes mellitus and hypertension represent the most common comorbidities no study has been done to determine the anthropometric indices as determinants of resistant Hypertension compared to conventional hypertension among type 2 diabetes patients. Resistant hypertension has been defined as high blood pressure (that is, blood pressure >140/90 mm Hg) despite therapy with at least three antihypertensive drugs; one being a diuretic at tolerated doses^2^.

Resistant hypertension has also been reported to be relatively common among type 2 diabetes patients. Importantly, the prevalence of diabetes has been increasing in the recent past^3^ indicating a future rise in the frequency of resistant hypertension. Among the hypertensives seen in general practice showed a prevalence of feasibly 5% while probably 50% in those seen by nephrologists^4^ while another study^5^, indicated that there was absence or even lack of data as regards the prevalence, incidence and prognosis of resistant hypertension. Therefore, there is a need for more studies to be done to find out the prevalence of resistant hypertension among type 2 diabetes and among other subsets of disorders/ diseases.

Obesity is a major contributor to preventive deaths in America today, and so these two conditions (obesity and overweight) pose greater health challenges^5^. This study 6, body mass index (BMI) levels associate with body fat as well as ensuing health risks and that future morbidity and death can be predicted by high levels of BMI. Obesity and its health risks can also be screened by BMI, although factors such as sex, ethnicity, age and muscle mass can influence the relationship between BMI and body fat. Therefore, the researcher chose to supplement BMI with waist hip ratio (WHR) to factor in the minor challenges that BMI does not address.

This study 7, recommended that WHR should habitually be used in clinical setting in addition to BMI to detect persons at high risk. Another study showed WHR as the best predictor for absolute Cardiovascular Heart Disease (CHD), diabetes, dyslipidemia risk in Torres Strait Islanders and Australian Aboriginal people. Incorporating WHR into routine health examinations in Australian indigenous people will enhance the evaluation of CVD risk^8^. Taking this into account, anthropometric indices could be some of the roots for resistant hypertension (RHTN) among type 2 diabetes (T2D) cases. Thus, the aim of this study was to determine the association of anthropometric indices with resistant hypertension among type 2 diabetes patients attending Kisii Teaching and Referral Hospital in Kenya

## Materials and Methods

### Study population

The study was carried out at Kisii Teaching & referral Hospital diabetic (special) clinic. Kisii Teaching and referral hospital is a level 6 Hospital that provides health service to the region that spans from western Kenya and South Nyanza of Kenya counties. The study participants included patients diagnosed with resistant hypertension and who are also type 2 diabetes patients attending the hospital as the study group while type 2 diabetes patients with non-resistant hypertension were included as the control group.

### Ethical Consideration

Ethical approval was obtained from the University of Eastern Africa, Baraton. Informed consent was sought from participants and they were free to withdraw at any level of their choice; their service delivery was not to be affected because they had withdrawn. The information collected was kept in confidence (i.e., the database had a key known only to the researcher). The participants got relevant information on how to manage their disorders and to prevent and/or delay disease-related complications. This research was not to risk any participant or cause any harm to the participants.

A proportionate sample size determination formula 9 was used to determine the sample size for the two groups (Diabetes/ Hypertension cases and Diabetes/ resistant hypertension).

n= {Z_1-α/2_√ [2P (1-P)] + Z_1-β_√ [P_1_ (1- P_1_+P_2_ (1- P_2_)]}^2^/ (P1-P2)^2^

**Figure:**
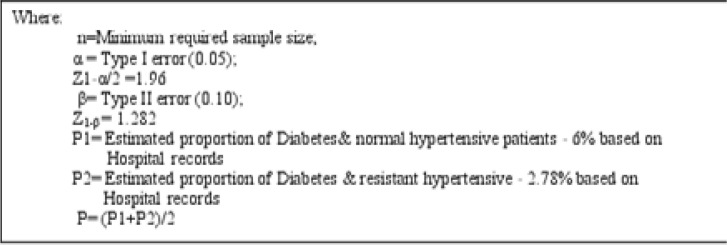


When substituted

n = {1.960√ [2(0.439) (0.561)] + 1.282√ [0.6(0.4) + 0.278 (0.722)]} 2/ (0.6- 0.278)2 = 47.8181

The minimum required sample size is therefore 47.8 = 48 study participants for each group making a total of 96 participants for the two groups.

The study used descriptive design, where forty-eight (48) study participants for each group making a total of 96 participants for the two groups were recruited consecutively at the diabetic clinics. Eligible participants in terms of age and who were willing to participate in the study were included in the study. Informed consent was obtained from the study participants. Participants who had poor nutritional feeding and were not adhering to drugs schedule and dosage after diagnosis with resistant hypertension were excluded from the study. The prospective participants were given adequate opportunities to discuss and contemplate their participation. Participants were also given time to ask questions and to have all concerns addressed. Throughout the study, free and informed consent process was exercised.

### Data collection

Structured questionnaires were used to collect anthropometric indices (BMI and WHR). Cases of resistant hypertension and type 2 diabetes were ascertained by a physician and if the respondent was treated for this in the last 12 months. The questionnaire included sections on height, weight, hip circumference and waist circumference. The questionnaires were distributed at recruitment to ensure that data was collected according to schedule. Body mass index involved taking of height and weight while waist hip ratio involved taking waist circumference and hip circumference. Diagnosis of diabetes was performed using various tests which oral glucose tolerance test with a 2-h value of 200 mg/dL or more, a random plasma glucose of 200 mg/dL or more with symptoms of diabetes, or a fasting plasma glucose of 126 mg/dL or more on more than one occasion 10. For hypertension, at a sitting position, blood pressure was measured 2 times on the left arm. The first measurement was made after at least 5 min rest and the second measurement was taken after 2 minutes interval 11.

Calculating body mass index was done by taking a person's weight, in kilograms, divided by their height, in meters squared, that is BMI = weight (in kg)/ height^2 (in m^2) 12. For adults aged 20 years and older, median BMI is on average of 25. 5 kg/m2. Weight and Median stature for men are 80 and 175. 5 cm. 0 kg while for women are 65. 6 kg and 161. 6 cm respectively 13. Waist Hip Ratio (WHR) was calculated by taking the Waist Circumference divided by the Hips Circumference 11. The optimal cutoff values of WHR are considered 0.82 for women and as 0.89 for men 14. The completed questionnaires were cross-checked for completeness of data and filling of the questions. Research assistants were trained adequately to ensure accuracy in reporting.

### Data analysis

Data was entered and analysed using STATA version 13 (StataCorp, Timberlake, UK). Categorical variables were summarized as frequencies and corresponding percentages. The test of hypotheses between categorical variables was done using Pearson's Chi-Square test while binary logistic regression and odds ratio was used to determine the relationship and association of the study variables. P-values ≤0.05 were considered statistically significant. The data were presented in tables and a logistic model was fitted and odds ratio of getting resistant hypertension determined.

## Results

The anthropometric indices of the study participants at recruitment were collected and summarized in tables 1–3. Forty-eight (48) conventional hypertension cases and an equal number of resistant hypertension participants among type 2 diabetes patients were used as the study participants. To determine the anthropometric indices as determinants of resistant hypertension compared to conventional hypertension among type 2 diabetes cases, the study evaluated the body mass index (BMI) and the waist hip ratio (WHR) of the respondents.

**Table 1:** Anthropometric Indices determinants of resistant hypertension compared to Conventional hypertension among type 2 diabetes patients

Study Variable reported in mean (SD)		Type of hypertension		P-Value
		Conventional Hypertension	Resistant Hypertension	
Anthropometric Indices	Body Mass Index (BMI)	27.2 (4.1)	29.3 (3.8)	**0.014**
	Waist- Hips-Ratio (WHR)	1.0 (0.113)	0.99 (0.119)	0.393

**Table legend:** BMI showed a mean of 29.3 and standard deviation of 3.8 among the study participants who had resistant hypertension while those who had conventional hypertension had a mean of 27.2 and a standard deviation of 4.1. Therefore, the null hypothesis that the Body Mass Index (BMI) is not a determinant of resistant hypertension compared with conventional hypertension among type 2 diabetes patients were rejected (P=0.014) an indication that the higher the BMI the more likely the patient will develop resistant hypertension. The waist-hip ratio for conventional hypertension had a mean of 1.0 and a standard deviation of 0.113 while mean and standard deviation of resistant hypertension was 0.99 and 0.119 respectively. In testing the null hypothesis that waist-hip ratio is not a determinant of resistant hypertension compared with conventional hypertension was accepted (P=0.393). These results indicate that the increase of WHR will not necessarily increase the chances of acquiring resistant hypertension.

**Table 2:** Logistic Regression Model Summary

-2 Log likelihood	Cox & Snell R Square	Nagelkerke R Square
123.068^a^	.099	.132

**Table legend:** The extent to which the independent variables (BMI and waist-hip-ratio) can change the dependent variable (resistant hypertension) can be explained to vary statistically by ranges between 9.9% to 13.2%.

**Table 3:** The Odds Ratio and logistic regression equation for Type of hypertension and Anthropometric indices

Variable	Risk Estimate (Odds ratio)				Logistic Regression Equation	
		Value	95% Confidence Interval		Wald test	Sig
			Lower	Upper		
Waist-hip ratio	Odds Ratio for Type of hypertension (Resistant/ Conventional)	1.352	0.459	3.983	0.299	0.585
	For cohort Waist -Hips Ratio = Normal range	1.286	0.521	3.172		
	For cohort Waist -Hips Ratio = Not Normal range	0.951	0.795	1.138		
	N of Valid Cases	96				
Body mass index	Odds Ratio for Type of hypertension (Resistant/ Conventional)	0.365	0.139	0.955	4.220	**0.040**
	For cohort BMI = 24.9 and below	0.471	0.225	0.985		
	For cohort BMI = Above 24.9	1.290	1.010	1.648		
	N of Valid Cases	96				

**Table legend:** BMI (sig=0.040) added significantly to the model/prediction but waist-hip-ratio (sig.=0.585) did not add significant to the model/prediction while according to the same table 11. The likelihood of an individual to suffer from resistant hypertension with BMI above 24.9 is higher (OR=1.290) as compared a BMI of 24.9 and below (OR= 0.471). Interestingly, the odds ratio for waist-hip ratio in the normal range was higher (OR=1.286) in causing resistant hypertension compared to the abnormal waist-hips ratio (OR= 0.951).

This study measured BMI (Body Mass Index) and Waist-Hip-Ratio (WHR) as anthropometric indices in the study participants and analysed their contribution to the prevalence of resistant hypertension among type 2 diabetes patients as presented in Table 1 below.

Results for BMI showed a mean of 29.3 and standard deviation of 3.8 among the study participants who had resistant hypertension while those who had conventional hypertension had a mean of 27.2 and a standard deviation of 4.1. Therefore, the null hypothesis that the Body Mass Index (BMI) is not a determinant of resistant hypertension compared with conventional hypertension among type 2 diabetes patients was rejected (P=0.014) an indication that the higher the BMI the more likely the patient will develop resistant hypertension. The waist-hip ratio for conventional hypertension had a mean of 1.0 and a standard deviation of 0.113 while mean and standard deviation of resistant hypertension was 0.99 and 0.119 respectively. In testing the null hypothesis that waist-hip ratio is not a determinant of resistant hypertension compared with conventional hypertension was accepted (P=0.393). These results indicate that the increase of WHR will not necessarily increase the chances of acquiring resistant hypertension.

Logistic regression model was also performed to ascertain the effects of anthropometric indices on the likelihood that participants have resistant hypertension compared to conventional hypertension among type 2 diabetes patients. The logistic regression modeling was done using stepwise and block wise. Based on Cox & Snell R Square and Nagelkerke R Square values, the extent to which BMI and waist-hip-ratio can change resistant hypertension has been shown to vary statistically by ranges between 9.9% to 13.20% as shown in table 2.

In order to determine the contribution of the BMI and waist-hip-ratio to the model and its statistical significance; odds ratio and a logistic regression equation was determined as shown in table 3. Wald test was used to determine the statistical significance of each of the variables. The statistical significance test which is found in the “Sig.” column indicated statistical contribution to the regression model. When logistic regression was performed, Wald test in the equation indicated that BMI (sig=0.040) added significantly to the model/prediction but waist-hip-ratio (sig=0.585) did not add significant to the model/prediction while according to the same table 11. The likelihood of an individual to suffer from resistant hypertension with a BMI above 24.9 is higher (OR=1.290) as compared a BMI of 24.9 and below (OR= 0.471). Interestingly, the odds ratio for waist-hip ratio in the normal range was higher (OR=1.286) in causing resistant hypertension compared to the abnormal waist-hips ratio (OR= 0.951).

## Discussion

Anthropometric indices are also identified as significant predictors of the likelihood of a patient with diabetes also presenting with hypertension or resistant hypertension if one is obese or has a high waist-hip ratio according to 15. A high BMI above 25 and WHR above 0.9 for men and 0.82 for females increase the risk of resistant hypertension among patients with type 2 diabetes. This outcome which is consistent with 16, 17 and 18 mean that persons who are obese are at a higher risk of suffering from resistant hypertension.

The association was positive and fairly strong among the participants in this study, where it was observed that higher BMI is more likely to cause resistant hypertension. Therefore, BMI could continuously be used to predict the occurrence of diabetes, hypertension and also resistant hypertension as supported by this study and others. Although some studies 7 find BMI as a strong predictor for obesity, hypertension and other cardiovascular diseases, others contrast by noting that BMI does not include measurement of other factors such as body fat. In some studies 14, the waist-hip ratio was the best predictor for type 2 diabetes in the study sample and in another study patients of resistant hypertension with high BMI and diabetes have an increased risk complication events compared to non-resistant hypertensive patients 17. Study findings among the Taiwanese population indicated WHR as a good anthropometric index for predicting the risk of type 2 diabetes, and optimal cut-off values of WHR being valued as 0.89 for men and 0.82 for women 16. In contrast, this present study showed waist-hip ratio is not a determinant of resistant hypertension compared with conventional hypertension. These results indicate that the increase of WHR will not necessarily increase the chances of acquiring resistant hypertension.

Overall, the present study expands our understanding of the anthropometric indices risk factors of resistant hypertension in diabetes patients. The study will also help the policymakers in formulating new policies and guidelines towards achieving better management and/or prevention of resistant hypertension and type 2 diabetes.

## Conclusion

Tests on data from patients indicated that anthropometric indices BMI also relate positively and fairly strongly to the occurrence of hypertension among patients visiting KTHR. Therefore, BMI shares a good indicator of whether a patient is likely to suffer resistant hypertension. Consequently, hypothesis that tested the association between anthropometric indices and resistant hypertension among type 2 diabetes patients attending Kisii Teaching and Referral Hospital) is partly rejected with a BMI P-value (0.014) and partly accepted with WHR having a P-value (0.393). These results indicate that the increase of WHR will not necessarily increase the chances of acquiring resistant hypertension.

## Recommendations

The present study, recommends broader and comprehensive health promotion programs to encourage and promote lifestyle modification and since limited information is available on determinants of resistant hypertension among type 2 diabetes patients, more studies with more control arms should be carried out to give more insight on resistant hypertension.
